# Effect of Sugars on Artemisinin Production in *Artemisia annua* L.: Transcription and Metabolite Measurements

**DOI:** 10.3390/molecules15042302

**Published:** 2010-03-30

**Authors:** Patrick R. Arsenault, Daniel R. Vail, Kristin K. Wobbe, Pamela J. Weathers

**Affiliations:** 1 Department of Biology and Biotechnology, Worcester Polytechnic Institute, WPI, 100 Institute Rd. Worcester MA 01609, USA; 2 Arkansas Bioscience Institute, Arkansas State University, Jonesboro AR, 72401 USA; E-Mail: danrvail@gmail.com (D.R.V.); 3 Department of Chemistry and Biochemistry, Worcester Polytechnic Institute, Worcester MA, 01609, USA; E-Mail: kwobbe@wpi.edu (K.K.W.)

**Keywords:** terpene, sugar signal, secondary metabolism

## Abstract

The biosynthesis of the valuable sesquiterpene anti-malarial, artemisinin, is known to respond to exogenous sugar concentrations. Here young *Artemisia annua* L. seedlings (strain YU) were used to measure the transcripts of six key genes in artemisinin biosynthesis in response to growth on sucrose, glucose, or fructose. The measured genes are: from the cytosolic arm of terpene biosynthesis, 3-hydroxy-3-methyl-glutaryl-CoA reductase (HMGR), farnesyl disphosphate (FPS); from the plastid arm of terpene biosynthesis, 1-deoxyxylulose-5-phosphate synthase (DXS), 1-deoxyxylulouse 5-phosphate reductoisomerase (DXR); from the dedicated artemisinin pathway amorpha-4,11-diene synthase (ADS), and the P450, CYP71AV1 (CYP). Changes in intracellular concentrations of artemisinin (AN) and its precursors, dihydroartemisinic acid (DHAA), artemisinic acid (AA), and arteannuin B (AB) were also measured in response to these three sugars. *FPS, DXS, DXR, ADS* and *CYP* transcript levels increased after growth in glucose, but not fructose. However, the kinetics of these transcripts over 14 days was very different. AN levels were significantly increased in glucose-fed seedlings, while levels in fructose-fed seedlings were inhibited; in both conditions this response was only observed for 2 days after which AN was undetectable until day 14. In contrast to AN, on day 1 AB levels doubled in seedlings grown in fructose compared to those grown in glucose. Results showed that transcript level was often negatively correlated with the observed metabolite concentrations. When seedlings were gown in increasing levels of AN, some evidence of a feedback mechanism emerged, but mainly in the inhibition of AA production. Together these results show the complex interplay of exogenous sugars on the biosynthesis of artemisinin in young *A. annua* seedlings.

## Introduction

**Figure 1:**
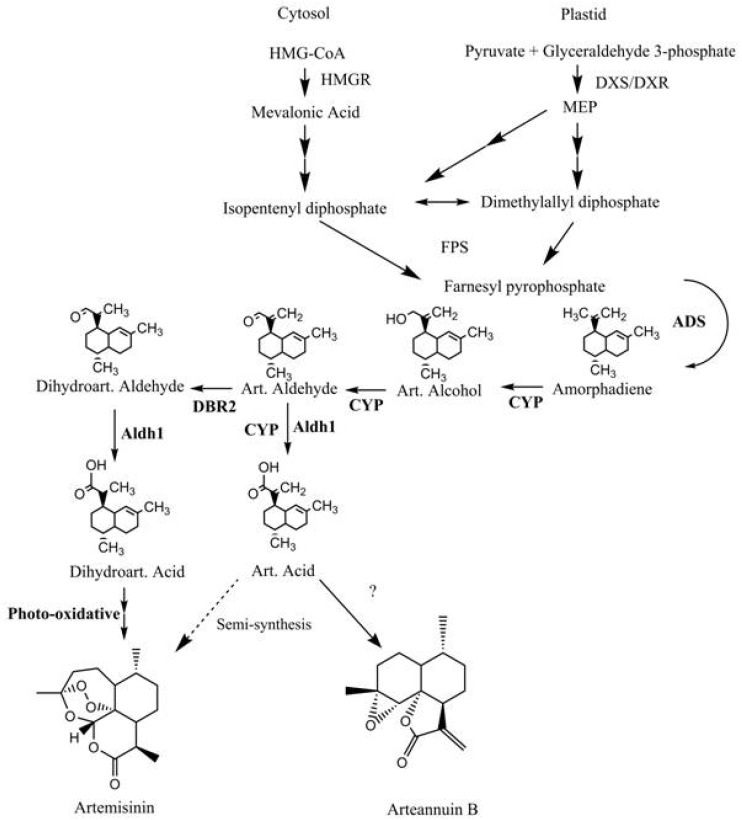
Artemisinin biosynthetic pathway. ADS, amorphadiene synthase; Aldh1, aldehyde dehydrogenase 1; CYP, CYP71AV1; DBR2, double bond reductase 2; DMAPP, dimethylallyl diphosphate; DXS, 1-deoxyxylulose 5- phosphate synthase; DXR, 1-deoxyxylulouse 5-phosphate reductoisomerase; HMGR, 3-hydroxy-3-methylglutaryl-CoA reductase; IPP, isopentenyl diphosphate; MEP, methyl erythritol phosphate pathway; MVA, mevalonic acid pathway.

Artemisinin combination therapy (ACT) is the most effective means of treating malaria [1]. Artemisinin (AN; Figure 1) is a sesquiterpene lactone that is produced by the plant *Artemisia annua* L. The plant has been part of traditional Chinese medicine for >2,000 years and used for a variety of ailments [2]. AN may also be an effective treatment for other health problems including those caused by cytomegalovirus, hepatitis B [3], schistosomiasis [4], and a variety of neoplasms [5]. Production *in planta* has been characteristically low and synthetic production is not yet economically feasible [6]. Various attempts at increasing production have yielded some positive results but because the control of AN biosynthesis *in planta* is largely not understood, regulation of this terpenoid still requires considerable investigation. To our knowledge this is the first report showing kinetic changes in transcription of the genes in the AN biosynthetic pathway in *A. annua* in response to sugars.

AN, one of a diverse pool of secondary metabolites produced by *Artemisia annua*, is produced by the glandular trichomes and sequestered in their epicuticular sacs [7,8]. AN is produced through the condensation and oxidation of three isopentenyl diphosphate (IPP) precursor molecules [7,8]. IPP is formed *via* either the cytosolic MVA pathway or the plastidic MEP pathway and evidence suggests that both pathways may be able to supply the IPP necessary for AN production [9]. Indeed, it seems that transfer of IPP between the two pools may be a significant factor in the production of other isoprenoids [10,11,12]. The condensation of three IPP molecules catalyzed by FDP synthase (FPS) first produces farnesyl disphosphate (FDP); then a sesquiterpene cyclase, amorphadiene synthase (ADS), catalyzes the formation of amorpha-4,11-diene [7,13,14,15]. A cytochrome P450, CYP71AV1 (CYP), catalyzes the next three reactions: oxidation of amorpha-4,11-diene to artemisinic aldehyde and also to artemisinic acid (AA) [16], which is then converted by a double-bond reductase (Dbr2) to dihydroartemisinic aldehyde, the presumed precursor to dihydroartemisinic acid (DHAA) [17]. The conversion of DHAA to AN has been shown to occur *in vitro* as a photo-oxidative reaction, though this may not necessarily be occurring *in vivo* [18]. Whatever the mechanism, the reaction involves the addition of three oxygen atoms and the formation of the endoperoxide pharmacophore of AN [19]. How the activity of these enzymes is controlled and how each influences the levels of AN or its precursors is not entirely known.

Numerous primary and secondary metabolites have been shown to be sugar responsive including products of the glyoxylate cycle [20], anthocyanins [21], and artemisinin [22]. In *Vitis* and more recently in *Arabidopsis*, a number of anthocyanin genes have been shown to be upregulated in response to sucrose [23,24]. The mechanism whereby these changes occur, however, is not entirely known and likely combines several different routes. Sugars may be acting as signaling molecules in plants through their interaction with protein receptors or through downstream by-products of their catabolism. Among the known sugar signal transduction pathways, the best characterized is the hexokinase dependent pathway, in which the phosphorylation of intracellular glucose limits its routes for metabolism and causes changes in gene transcription through the degradation of transcription factors [25]. A key example of this is the glucose dependant degradation of the EIN3 transcription factor that is mediated through hexokinase 1 (HXK1), making the presence of glucose antagonistic to the action of ethylene in *Arabidopsis* [26]. There also exist certain disaccharide specific sensing mechanisms that, through the modulation of translation, play a role in sugar-specific metabolic processes [27].

It can be difficult to determine the direct effect of sugars since not only is there significant crosstalk in sugar signals, the action of invertases readily converts sucrose to its component monosaccharides, glucose and fructose, further confounding interpretation of much signaling pathway work [25,28]. While it seems that in some cases sugars may perform their roles and modulate metabolism independently of one another, there are many more examples of various sugars acting synergistically or antagonistically with one another with diverse consequences of these interactions. For instance, in *Arabidopsis* sucrose has been shown to accelerate flowering [29] while glucose has been shown to significantly delay flowering [30].

In *A. annua* sugars were shown to significantly alter the levels of AN production when seedlings were grown on equimolar carbon supplied in the form of either sucrose, glucose, or fructose [22]. Furthermore, when grown in sucrose-free medium, artemisinin levels were directly proportional to glucose as the ratio of glucose to fructose was increased from 0 to 100%. Here we report that sugar composition not only affects the levels of AN and its related metabolites but also the levels of AN specific gene expression. From these results it is becoming apparent that sugars function in AN biosynthesis not only as carbon sources but also as potential signals for the induction or repression of key AN biosynthetic genes. What is not clear is the mechanism whereby these changes in gene expression are acting to affect the level of artemisinin related metabolites. 

## Results and Discussion

### Changes in relative gene expression

To determine if sugars were affecting the transcription of genes in the artemisinic biosynthetic pathways, we used Real-Time PCR to measure the relative transcript levels of the following genes: HMGR DXS, DXR, FPS, ADS, and CYP. Similar to methods used in sugar signaling studies in Arabidopsis, transcript levels were measured in seedlings grown in all three sugars with equimolar amounts of carbon. Then the responses in glucose and fructose were compared to transcript levels from seedlings grown in sucrose. 

**Figure 2:**
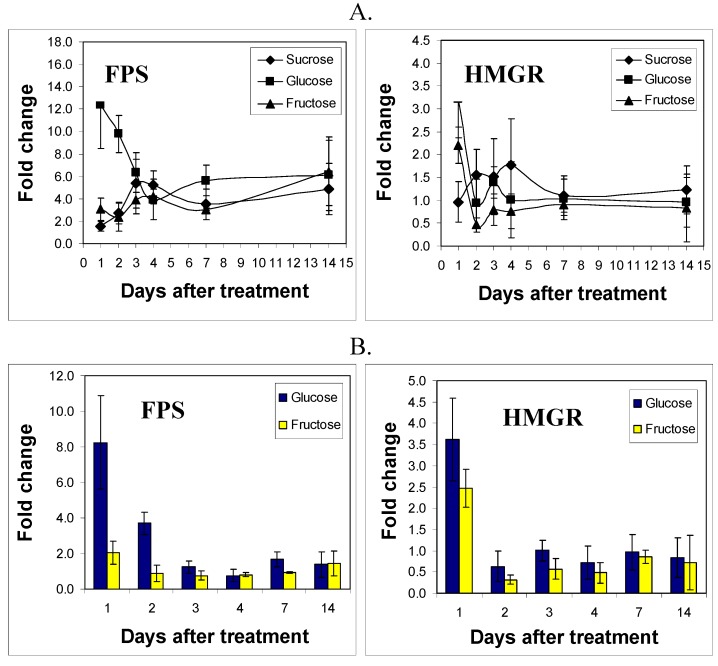
(A) Changes in HMGR and FPS in *A. annua* seedlings grown 14 days in glucose, fructose, or sucrose relative to day zero. (B) Relative response of HMGR and FPS in *A. annua* seedlings grown in glucose or fructose and compared to growth in sucrose. Bars are ± SD.

The early pathway genes, HMGR and FPS, showed differences in expression soon after exposure to the test sugar (Figure 2A). Compared to those fed sucrose, seedlings that were fed glucose or fructose showed only a slight stimulation in HMGR transcripts after 1 day (3.5 and 2.5 fold, respectively) (Figure 2B). In contrast to growth in fructose, FPS transcript levels in glucose-fed seedlings were more than 8 fold greater than in sucrose-fed seedlings at day 1 (Figure 2B). Both FPS and HMGR transcript levels declined in both glucose and fructose-fed seedlings by day 3, and by day 14 mRNA levels were identical regardless of sugar composition (Figure 2B). 

**Figure 3:**
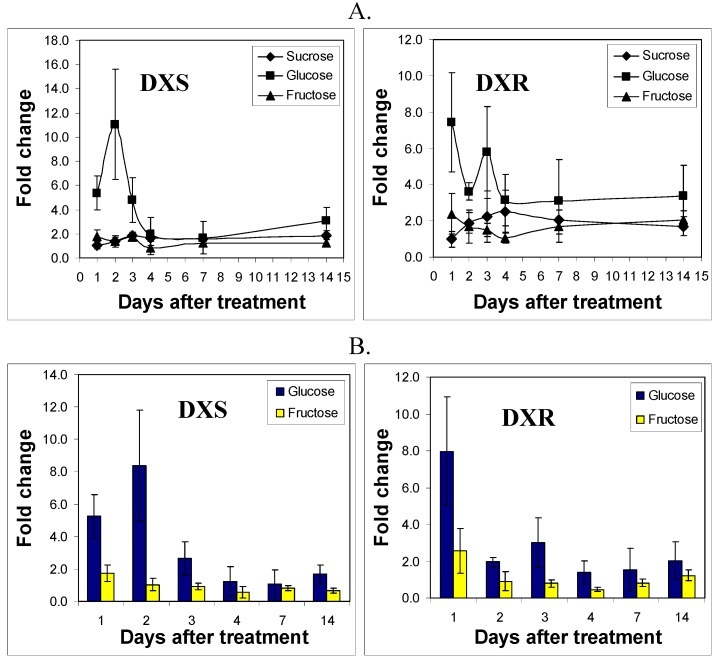
(A) Changes in DXS and DXR in *A. annua* seedlings grown 14 days in glucose, fructose, or sucrose relative to day zero. (B) Relative response of DXS and DXR in *A. annua* seedlings grown in glucose or fructose and compared to growth in sucrose. Bars are ± SD.

Glucose also stimulated both DXS and DXR from the MEP pathway (Figure 3A). Compared to transcript levels at inoculation (day 0), DXS and DXR increased approximately 5 and 7 fold, respectively, in seedlings fed glucose for one day. While the level of DXS increased further at day 2, the level of DXR transcripts dropped by 50%. After 2 days DXS transcripts also declined and remained low. On the other hand, DXR transcripts rose again on Day 3 to about 6 fold greater than at inoculation (Figure 3A), but then declined, remaining steady at 3 fold greater than day 0 until harvest at day 14. 

Compared to growth in sucrose, only glucose-fed seedlings showed significant responses in their DXS and DXR transcript levels (Figure 3B), essentially paralleling the data relative to day zero. Fructose-fed seedlings, however, showed no significant changes in either DXS or DXR transcript levels when compared to seedlings fed sucrose (Figure 3B). 

**Figure 4:**
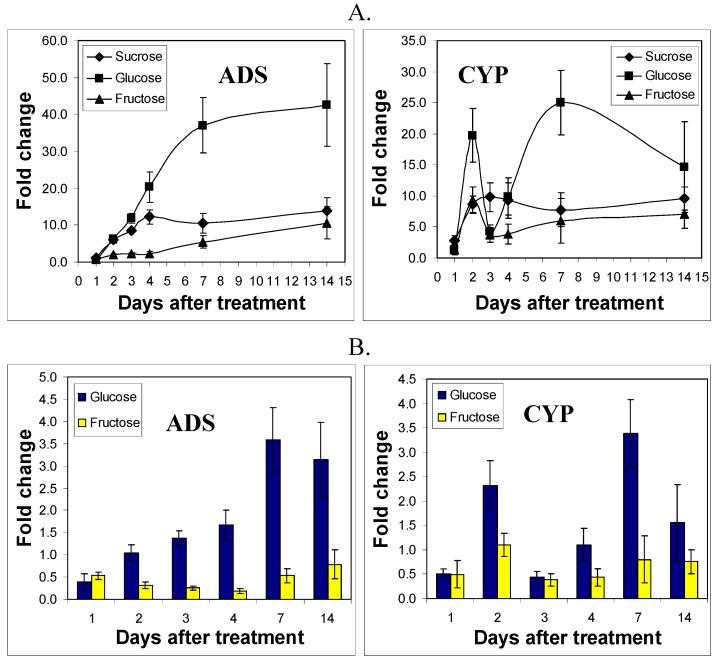
(A) Changes in ADS and CYP in *A. annua* seedlings grown 14 days in glucose, fructose, or sucrose relative to day zero. (B) Relative response of ADS and CYP in *A. annua* seedlings grown in glucose or fructose and compared to growth in sucrose. Bars are ± SD.

In contrast to the other 4 genes, both ADS and CYP responded quite differently to the three sugars tested (Figure 4A). While both showed increases in steady state levels of mRNAs over the time course of the experiment, glucose-fed seedlings showed greater increase in ADS and CYP transcripts than did either sucrose or fructose-fed seedlings. This increase was especially prevalent for ADS at days 4-14 when levels in glucose-fed seedlings were as much as 7 fold higher than in seedlings grown in fructose, and triple that of seedlings grown in sucrose. Relative to day 0, ADS expression levels in glucose-grown seedlings increased rather steadily from day 0 to day 7 reaching nearly a 40-fold increase when harvested at day 14 (Figure 4A). ADS increases in fructose and sucrose- fed seedlings were less dramatic, but each still showed a significant 10-fold increase at day 14 when compared to day 0.

The CYP mRNA levels were more variable (Figure 4A). Again, maximum expression was seen in glucose-fed seedlings, but in contrast to the steady increase observed for ADS, there appeared to be a biphasic expression pattern, with peaks at day 2 (20x) and day 7 (25x). Although far less prominent, this biphasic behavior also seemed to appear in the data from fructose-fed seedlings (Figure 4A). 

Compared to the sucrose-fed seedlings, only glucose-fed seedlings showed any significant changes in ADS and CYP (Figure 4B). The level of ADS transcripts steadily increased to about 3 fold that of sucrose, while CYP transcript levels showedtwo peaks at days 2 and 7 (Figure 4B).

### Changes in artemisinic metabolite levels

The amounts of artemisinin and the artemisinic metabolites, AA, AB, and DHAA, were also analyzed in these same tissues. DHAA is thought to be a direct precursor of AN, while AA and AB are also formed from the product of the ADS gene, but apparently through a distinct pathway (Figure 1). All are products that appear after CYP in the biosynthetic pathway, so their presence depends on all the genes tested. 

**Figure 5:**
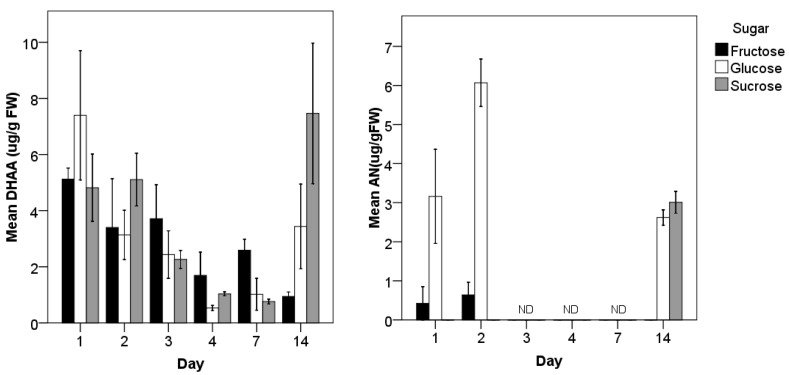
AN and DHAA levels in *A. annua* seedlings grown 14 days in glucose, fructose, or sucrose. Bars are SE.

**Figure 6:**
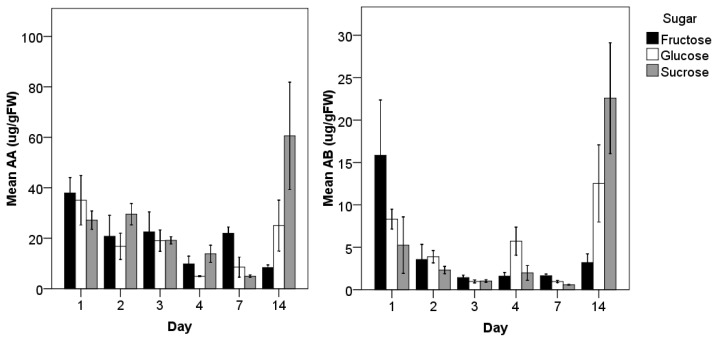
AA and AB levels in *A. annua* seedlings grown 14 days in glucose, fructose, or sucrose. Bars are SE.

There is significantly more AA and AB than either AN or DHAA indicating that this particular cultivar (YU, Yugoslavian origin) of *A. annua* is the AA/AB and not the AN/DHAA chemotype (Figure 5 and Figure 6). In contrast to the mRNA transcript data, there was a general decline between day 1 and day 7 for AA, AB and DHAA regardless of the carbon source in the medium (Figure 5 and Figure 6). At day 14, however, there was a different outcome; in both glucose and sucrose-fed seedlings, these compounds showed an increase, which was strongest for sucrose media. In fructose-fed seedlings, however, there was no significant increase in any of these compounds at day 14. 

AN levels showed a very different pattern of production compared to the other metabolites and, interestingly, were also reduced to levels below the limits of detection from days 3 through 7 suggesting that as the seedlings begin to develop true leaves, AN may be further metabolized (Figure 5). Consistent with early cytosolic and plastidic terpenoid pathway gene expression data, glucose stimulated increases in AN yield at day 1 and 2. Compared to those fed fructose, seedlings fed glucose produced about 8 fold more AN at day 2. However, by day 14 there was no difference in AN yields between glucose and sucrose-grown plants; AN from fructose-fed seedlings was undetectable (Figure 5). 

Consistent with its role as an AN precursor, DHAA levels declined in glucose fed-seedlings between day 1 and 2 as AN increased (Figure 5). At day 14, however both DHAA and AN were at approximately the same level. In contrast, in fructose-fed seedlings DHAA steadily declined in concentration and the AN concentration dropped rapidly to undetectable levels, consistent with an inhibitory effect of fructose on AN biosynthesis. Although AA is the apparent precursor to AB, AA does not show expected precursor kinetic patterns (Figure 6). In glucose-grown seedlings, both AA and AB declined, until day 14 where AA decreased in concentration about as much as AB increased. In fructose-fed seedlings, however, the AA concentration remained much higher than that of AB, which plummeted to a low level soon after transfer into fructose and remained at a low level until harvest (Figure 6).

### Response of seedlings to exogenous AN

**Figure 7:**
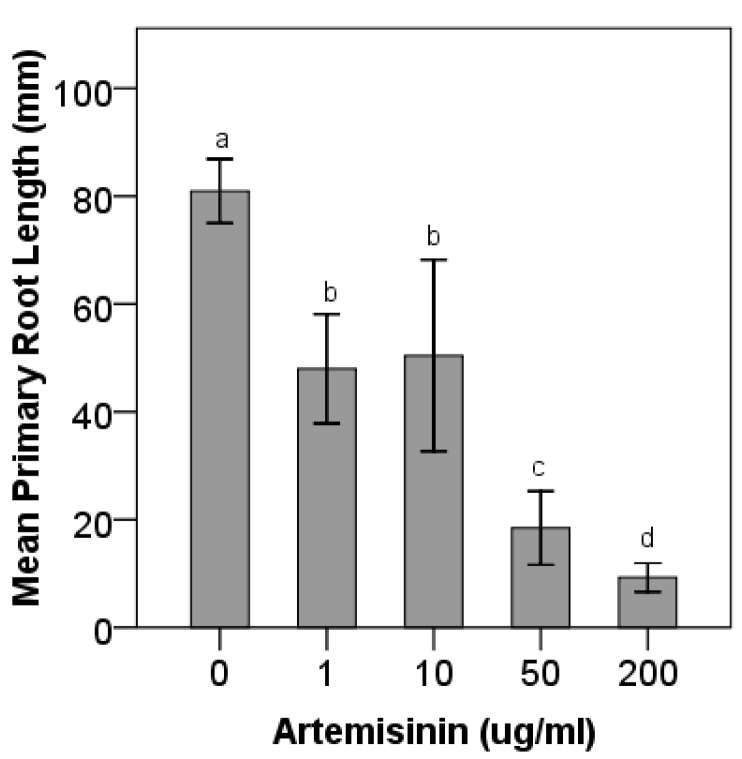
Root growth of *A. annua* seedlings grown in different concentrations of artemisinin. Letters indicate statistical differences at p ≤ 0.05(*via* ANOVA/Tukey HSD).

To determine if there is a possible feed-back mechanism in response to exogenous AN, it was added to the culture medium to determine its effect on growth and metabolite concentrations of young seedlings. When increasing concentrations of AN were added to seedlings, root elongation was inhibited and was dose dependant (Figure 7). Seedlings treated with high concentrations of AN (200 µg mL^-1^) showed an 8 fold reduction in the mean length of their primary roots after 10 days of exposure (Figure 7). Even seedlings grown in relatively low concentrations (1 µg mL-1) showed >40% reduction in mean root length. When the concentrations of AN and AN related metabolites were measured in the leaves and cotyledons of seedlings incubated in AN, after 10 days, AN was detectable in all tissues at levels similar to untreated controls, however it was not clear if AN was produced endogenously. More interestingly, at concentrations ≥50 µg mL^-1^ AN, endogenous levels of AA fell to below detectable limits. These preliminary results suggest that AN may be acting to regulate the levels of AA as part of a putative feedback loop.

### Correlation between transcript and metabolite levels

Our results clearly show differential responses in both transcript level and artemisinic metabolite abundance in response to exogenous sugar composition. The most intriguing results are in relation to both ADS and CYP transcript abundance. These two genes represent committed steps towards the measured artemisinic metabolites. Although there are no direct correlations between the transcript levels of these two genes and metabolite abundance, they are both upregulated by glucose, and repressed by fructose. Thus, the data suggest that the interplay between transcript abundance and product formation is more complicated than previously hypothesized.

In examining ADS transcript abundance in relation to the sum totals of all artemisinic metabolites measured, we observe what may be a negative correlation. This may be indicative of a feedback mechanism that is attempting to regulate the levels of artemisinic metabolites in the plant. Since AN is phytotoxic even to *A. annua* [31], it is not surprising that mechanisms would exist to control its production, especially in young seedlings. Indeed, *A. annua* seedlings do not grow well when AN levels is added to the culture medium and the production of at least one artemisinic metabolite (AA) appears also to be inhibited (Figure 7). 

The results in this study suggest that fructose may be the specific signal that causes changes in transcript levels of ADS and CYP. When seedlings are exposed to sucrose, the sugar is inverted yielding an equivalent molecule each of glucose and fructose, thus only the glucose medium is actually free of fructose. Given that sucrose and fructose rarely caused major increases in expression level, it seems plausible that a fructose sensing mechanism may be working to repress the expression of these late pathway genes, for example, by action of a fructokinase or by a perturbation of the sucrose synthase pathway. Although glucose significantly stimulated transcript levels of both ADS and CYP, the differences observed in metabolite levels between sucrose and glucose were relatively minimal. This suggests that the level of fructose may be the more important determinant of observed metabolite levels. Concentrations of both AB and AN are rather rapidly and greatly reduced after seedlings are fed fructose, so inhibition of these pathways by fructose is plausible. An alternative hypothesis is that fructose stimulates the conversion of AN and AB to something else.

An earlier study by Wang and Weathers [22] showed that an increase in the ratio of glucose to fructose is reflected by a corresponding and proportional increase in AN yield. The relative amounts of AN we observed at the end of the 14 day growth period are, however, different from those reported earlier for the YU strain [22], which showed a 200% increase in AN levels in glucose compared to fructose and sucrose in similarly grown seedlings. These differences may be due to culture in liquid vs. in semi-solid media, or to differences in tissue extraction methods. In this study AN is extracted from fresh tissue instead of from dried tissue, the method used by Wang and Weathers [22]. Drying *A. annua* prior to extraction of AN significantly increases the amount of AN extracted from the tissues [32], so it is not possible to compare directly the AN measured using the two different methods. Furthermore, use of fresh tissue extracts provides a more valid comparison of pathway metabolites with gene transcripts levels; both are measured in real time. 

Although the data suggest that fructose is acting as the primary signal molecule for a transcriptional response, glucose in particular seems to cause increases in many of the gene transcripts in the biosynthetic pathway leading to AN. Therefore an alternative explanation for increased biosynthesis of this particular secondary metabolite in response to glucose may be that AN and AB are formed as DHAA and AA, respectively, and act as sinks for reactive oxygen species (ROS), specifically singlet oxygen [33]. AN has five oxygens in its molecular structure (Figure 1) including two that comprise the therapeutically important endoperoxide bridge [19]. The last steps in the biosynthesis of AN are considered to be photooxygenic, involving the spontaneous reaction of DHAA with singlet oxygen, a reaction identical to the photooxidation of polyunsaturated fatty acids [34]. This step effectively sequesters singlet oxygen in the AN structure and the plant then stores the ROS-AN in its trichomes. Experimental data from Kim *et al*. [35] provided evidence for a possible connection between oxygen and AN. They showed that highly aerated hairy root cultures of *A. annua* produced significantly more AN than poorly aerated cultures [35]. 

ROS are cytotoxic to cells, and singlet oxygen in particular, may act as an apoptogenic signal in plant cells; therefore, it is important that accumulation of these toxic molecules be minimized [36,37]. Soluble sugars and glucose in particular, through their metabolism, are sources of oxidative stress and plants have developed many evolutionary mechanisms to protect themselves from ROS [38,39]. It has been proposed that DHAA and AA may act as ROS sinks, and in this study modulation of the cellular redox environment by sugars may help to explain the observed changes in AN and AB levels. This hypothesis is further supported by our recent study where the AN concentration in *A. annua* tissues was significantly reduced in the presence of a ROS scavenger, ascorbic acid [40]. The difference in response to sucrose, glucose and fructose may be related to specific sugar signaling pathways and the relative abundance of each constituent monosaccharide. 

A primary source of ROS in plants is photosynthesis, which is intimately involved with sugar metabolism of the plant [41,42]. In addition, intracellular sugar composition has been shown to alter starch production rates and cellular redox states [43]. Thus, changes in ROS and anti-oxidant compounds with sugar composition would be expected here as well. It is also worthwhile to note that while AN metabolite levels steadily declined over the first 7 days in sucrose and glucose-fed seedlings, these both showed increases by day 14 while fructose-fed seedlings showed a decrease. Between 7 and 14 days, when transcript levels remained relatively stable, we may be observing the onset of photosynthetic activity or other major developmental changes occurring over the two weeks of seedling development. If the proposed role for AN as an oxygen sink is correct, then AN content should increase with an increased rate of photosynthesis, a major source of singlet oxygen and other ROS. We would expect significant sugar depletion in the medium by day 14; by this time true leaves are fully emerged, and the plants are likely deriving more of their metabolic carbon from the reduction of CO_2_ rather than from sugars in the medium. In plants grown in sucrose or glucose, AN content does increase during this time, which is consistent with this hypothesis.

Early cytosolic and plastidic pathway gene transcript levels seem to show little to no correlation to artemisinin precursor levels. At early time points, however, these do seem to correlate with relative AN levels. In the case of HMGR, when compared to sucrose-fed seedlings, both fructose and glucose cause an initial increase in transcript levels. This pattern of transcript levels is consistent with a possible sucrose specific, down-regulation of expression that is rapidly mitigated by the extracellular inversion of sucrose to glucose and fructose. The pattern of expression observed for FPS, DXS, and DXR, on the other hand, differed from HMGR in that differences between fructose and sucrose were minimal while the effects of glucose resulted in about an eight fold up-regulation in expression on day 1 (day 2 in the case of DXS). The patterns of expression observed for FPS, DXR, and DXS correlate most closely with AN production, while the AN specific genes show far less correlation, suggesting that, in seedlings, control of AN production *via* these later enzymes is not at the level of transcript abundance. 

In the case of FPS, these results appear consistent with other work indicating that *A. annua* plants expressing an additional copy of the FPS gene showed 3-4 fold higher levels of AN than their wild type counterparts [44]. Another study by Ma *et al*. [45], however, contradicted this conclusion. Their FPS over expressing F4 strain of *A. annua* produced less AN than the nontransgenic 001 line. 

After day 2, early pathway genes (HMGR, DXS, DXR, FPS) showed no significant differences in response to different sugars suggesting that sugar composition is not responsible for the transcriptional regulation of these genes later in development and that the level of transcriptional activation of these genes may have little to do with the levels of production of artemisinic metabolites. Indeed, the differences observed at early time points may simply be due to the movement from a sugar free medium to one of our experimental conditions. After the plants adjust to the difference they return to steady state levels of expression, indicating that the difference in transcript levels of the early pathway genes is in fact due to specific experimental conditions. The marked response in late pathway genes (ADS, CYP) further demonstrates the complex patterns of signaling interactions and highlights challenges inherent with measuring responses to sugars that may act as both signal and substrate molecule.

## Materials and Methods

### Seed sterilization and culture conditions

Seeds from the YU strain (cultivar YU, isolated from the former Yugoslavia, from Dr. Nancy Acton, Walter Reed Army Institute of Research, Silver Spring, MD) of *Artemisia annua* L. were used in all experiments. For experiments that provided individual sugars to plants, seeds were surface-sterilized and synchronously germinated according to the protocol developed by Wang and Weathers [22]. Seedlings were then inoculated in experimental media according to the protocol developed by Towler and Weathers [9]. Ten seedlings were placed in a 50 mL Erlenmeyer flask containing 5 mL autoclaved Gamborg’s B5 medium [46] at pH 5.7 to which filter-sterilized 3% (w/v) sucrose, glucose, or fructose was added. The flasks were placed on a rotary shaker at 100 rpm under continuous cool-white fluorescent light. Samples were harvested after 1, 2, 3, 4, 7, and 14 days of incubation. Three replicates (30 seedlings per replicate) were harvested at each time point for each sugar treatment.

In the case of artemisinin exposure, seedlings were sterilized as above and germinated to the two cotyledon stage. Germinated seedlings were placed into B5 media with 3% sucrose and augmented with 0-200 µg mL^-1^ AN. Due to solubility issues, AN was first dissolved in 70% ethanol before being serial diluted and added to media. The final concentration of ethanol in each flask was 0.35% and this amount was also added to controls. After 10 days growth under continuous light, seedlings were harvested, root lengths measured, and shoot tissues (cotyledons and true leaves) were extracted for artemisininc metabolite analyses. It was necessary to wait 10 days in order to obtain adequate amounts of shoot tissues for extraction.

### RNA isolation and real-time RT-PCR

Methods are briefly described here, but can be found in more detail in Mannan *et al*. [40]. After harvest, plant samples were immediately flash frozen in liquid nitrogen, ground, and RNA extracted and purified for reverse transcription to cDNA. Analysis was by Real-Time PCR using primers listed in Table 1 for the seven target genes: 3-hydroxy-3-methyl-glutaryl-CoA reductase (HMGR) from the cytosolic mevalonic acid dependant IPP pathway, 1-deoxyxylulose 5- phosphate synthase (DXS) and 1-deoxyxylulouse 5-phosphate reductoisomerase (DXR) from the plastidic mevalonate independent IPP pathway, and farnesyl diphosphate (FPS), amorpha-4,11-diene synthase (ADS), and the P450 cytochrome , CYP71AV1 (CYP); the 18S ribosomal small subunit gene was used as a normalizing factor. Primers were designed with PrimerSelect (Lasergene, DNAStar, Inc) using cDNA sequences specific for *Artemisia annua* available at NCBI. Relative fold changes in gene expression were calculated based on the 2^-ΔΔCT^ comparative method [47,48,49].

### Artemisinin and artemisinic precursor quantification

Using freshly harvested tissues, AN and its metabolites, AA, AB and DHAA, were extracted and measured by LC/MS as described in Mannan *et al*. [40]. 

**Table 1:** Primer sequences for target gene amplification by RT-PCR.

Gene	Direction	Sequence (5' => 3')	Base Pairs	Product Length
ADS	Forward	ATACAACGGGCACTAAAGCAACC	23	297 bp
ADS	Reverse	GAAAACTCTAGCCCGGGAATACTG	24	297 bp
CYP	Forward	GGGGTTAGGGATTTAGCCAGAA	22	218 bp
CYP	Reverse	AATTGCCTCCAGTACTCACCATAA	24	218 bp
DXR	Forward	ATTGCTGGCGGTCCCTTTGTTCTT	24	237 bp
DXR	Reverse	CTTTTCTCCCCATGCTCAGTTAGG	24	237 bp
DXS	Forward	ATGGGTTGGCGGGATTCAC	19	274 bp
DXS	Reverse	CCGTCAAGATTGGCAGTAGGTAAA	24	274 bp
FPS	Forward	GTATGATTGCTGCGAACGATGGA	23	211 bp
FPS	Reverse	CGGCGGTGAATAGACAATGAATAC	24	211 bp
HMGR	Forward	GGTCAGGATCCGGCCCAAAACATT	24	251 bp
HMGR	Reverse	CCAGCCAACACCGAACCAGCAACT	24	251 bp
18S	Forward	TCCGCCGGCACCTTATGAGAAATC	24	219 bp
18S	Reverse	CTAAGAACGGCCATGCACCACCAC	24	219 bp

Abbreviations: ADS, amorpha-4,11-diene synthase; CYP, P450 CYP71AV1; DXR, 1-deoxy-D-xylulose-5-phosphate reductoisomerase; DXS, deoxy-D-xylulose-5-phosphate synthase; FPS, farnesyl disphosphate synthase; HMGR, 3-hydroxy-3-methylglutaryl-CoA reductase; 18S, 18S ribosomal small subunit.

### Statistical analysis

All experiments were run in triplicate and fold change values were expressed as the mean ± SD. Results were averaged and compared against controls to determine statistical differences. Statistical analyses of real-time PCR data were performed using the non-parametric Mann-Whitney *U* test [50]. Statistically significant results are those with a P-value less than or equal to 0.05.

## Conclusions

This is the first study to show that sugars play a role in the accumulation of artemisinin related gene transcripts and in the production of artemisinic precursor molecules. While sugar composition clearly has profound effects on the transcript level of numerous key genes, how modulation of these transcript levels is integrated into the greater model of control of AN production is not clear. Also presented is preliminary evidence suggesting that AN may be acting as an inhibitor of its own biosynthesis. Clearly the role of sugars in AN production is complex and untangling it from confounding factors of growth and development is a challenge that remains. However, we have clearly shown that at least in *A. annua* seedlings, cues from sugars can have dramatic effects on the biosynthesis of artemisinin. Plant growth and metabolism are in constant flux with respect to sugars and these data could prove useful in furthering our fundamental understanding of the regulation of not only AN, but also other terpenes *in planta*.
